# Development of a Nomogram Model to Predict Mortality in ANCA‐Associated Vasculitis Patients With Pulmonary Involvement

**DOI:** 10.1111/crj.70067

**Published:** 2025-04-22

**Authors:** Qifang Guo, Yijia Shao, Le Yu, Xiuling Zhang, Jingjing Shang, Xueqin Feng, Wei Zhou, Xinwang Duan

**Affiliations:** ^1^ Department of Rheumatology and Immunology, Second Affiliated Hospital, Jiangxi Medical College Nanchang University Nanchang Jiangxi China

**Keywords:** ANCA‐associated vasculitis, clinical feature, nomogram, prognosis, pulmonary involvement

## Abstract

**Objective:**

Risk assessment and prognosis prediction are crucial for patients with pulmonary involvement in antineutrophil cytoplasimc antibody associated vasculitis (AAV). This study was conducted to create and internally validate a prognostic model for mortality of pulmonary involvement in patients with AAV that provides individualized risk assessments.

**Methods:**

A cohort of 150 patients diagnosed with AAV at the Second Affiliated Hospital of Nanchang University Hospital between January 2013 and July 2022 was included, using data obtained from the Chinese Rheumatism Data Center (CRDC). The model was developed using Cox proportional hazards regression and the least absolute shrinkage and selection operator. To validate the model, assessments were conducted for discrimination, calibration, and through decision curve analysis.

**Results:**

The mean survival time of lung involvement AAV patients was 57.0 ± 4.1 months. In the final predictive model for death, four clinical variables were included: age at baseline, history of tumors, baseline hemoglobin level, and the level of the percentage of forced vital capacity to the normal predicted value. One‐, two‐, and three‐year AAV patients with pulmonary involvement mortality probability‐predictive nomogram were established. Internal validation of the model was conducted, yielding Harrell's concordance index (0.884), a Brier score of 0.088, and a calibration curve indicating satisfactory performance.

**Conclusion:**

We constructed a risk model utilizing easily accessible clinical risk factors, which could accurately forecast the future mortality risk associated with pulmonary involvement in AAV patients.

## Graphical abstract

Risk assessment and prognosis prediction are vital for pulmonary involvement in AAV. Using data from 150 patients in the Chinese Rheumatism Data Center, we developed and internally validated a mortality prediction model. The final model included four clinical variables: baseline age, tumor history, hemoglobin level, and percentage of forced vital capacity to predicted value, demonstrating excellent predictive performance.

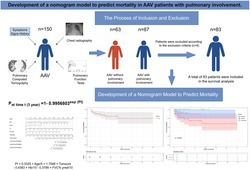

## 1 Introduction

Antineutrophil cytoplasmic antibody (ANCA) associated vasculitis (AAV) is a group of relatively rare autoimmune diseases characterized by necrotizing inflammation of the vascular wall, mainly involving small and medium‐sized vessels throughout the body. The classification of vasculitis, according to the 2012 revised International Chapel Hill Consensus Conference Nomenclature of Vasculitides (CHCC2012), includes microscopic polyangiitis (MPA), granulomatosis with polyangiitis (GPA), and eosinophilic granulomatosis with polyangiitis (EGPA) [1]. AAV attacks multiple organs and systems, including the respiratory system, the renal system, the mucocutaneous system, eyes, ears, nose, and the upper respiratory tract, with a wide range of clinical manifestations. Because of abundant vasculature, lung involvement can be the first manifestation of AAV patients [2, 3].

Pulmonary involvement is one of the hallmark lesions of AAV [4]. Previous studies have shown that the lung involvement is one of the independent predictors for mortality in patients with AAV and early lung injury can affect the prognosis of AAV patients [5, 6]. High‐resolution computed tomography (high‐resolution computed tomography, HRCT) is an important tool for assessing the pattern of lung involvement in patients. Abnormal findings are present in 52%–80% of AAV patients on HRCT, mainly manifested as interstitial lung disease (ILD), pulmonary granuloma (PG), alveolar hemorrhage (AH), and airway involvement (AI) [3, 7, 8]. The characteristics of the pulmonary involvement exhibit variability according to the specific type of vasculitis [9]. The presence of lung involvement in AAV patients affects patient prognosis and can lead to increased mortality rates [10, 11]. Patients with AAV in the presence of pulmonary involvement, such as alveolar hemorrhage and interstitial lung disease, tend to be more susceptible to death, which imposes significant social and economic costs. Despite the significance of promptly detecting mortality risk in AAV patients with lung involvement, there remains a paucity of evidence‐based screening strategy for them. Consequently, the clinical imperative to identify mortality risk at an earlier stage in AAV patients with pulmonary involvement remains strong. In the study, we respectively constructed a nomogram model to predict the future mortality risk associated with pulmonary involvement in AAV patients and then internally validated the model's performance using the bootstrap resampling method.

## 2 Patients and Methods

### 2.1 Participants

This was a prospective cohort study. A total of 150 patients diagnosed with AAV at the Second Affiliated Hospital of Nanchang University between January 2013 and July 2022 were prospectively recruited using data obtained from the Chinese Rheumatism Data Center (CRDC). All cases recruited in this study satisfied the AAV classification criteria of the 2012 Chapel Hill Conference and the diagnostic criteria established by the American College of Rheumatology (ACR) in 1990. The first admission served as the baseline measurement. Patients were excluded from the study based on the following exclusion criteria: (1) systemic lupus erythematosus, rheumatoid arthritis, allergic purpura, and other connective tissue diseases; (2) secondary vasculitis induced by medication; (3) tuberculosis and human immunodeficiency virus (HIV); and (4) other primary or secondary pulmonary conditions. All patients were thoroughly briefed and provided written‐informed consent before participating in this study. The study received approval and oversight from the ethics committee of the Second Affiliated Hospital of Nanchang University. (Approval Number 202212). Furthermore, this study complied with the requirements of the Helsinki Declaration.

### 2.2 Data Collection

We prospectively collected data at the initial admission and throughout subsequent follow‐up visits, including demographic characteristics, medical history, comorbidities, clinical characteristics, laboratory examinations, immunological indicators, biological markers, pulmonary computed tomography (CT) scan results, disease evaluation indicators, treatment plan, and treatment effectiveness. Serum ANCA levels were assessed using a combination of indirect immunofluorescence (IIF) and enzyme‐linked immunosorbent assay (ELISA). Throughout the entire follow‐up duration, we prospectively gathered and documented the efficacy of drug treatments as well as the survival status of the patients. Additionally, AAV patients with pulmonary involvement were categorized into four groups based on HRCT scan results: patients with interstitial lung disease (the ILD group), patients with pulmonary granuloma (the PG group), patients with alveolar hemorrhage alone (the AH group), and patients with airway involvement alone (the AI group). This division aimed to investigate potential associations between the type of lung involvement and patient prognosis.

### 2.3 Follow‐ups and definitions

Patients were regularly followed up either at the outpatient clinic or through recurrent inpatient admissions from January 2013 to July 2022. Comprehensive follow‐up assessments were scheduled and documented on a monthly basis during the initial 3 months for all patients, later every 3–12 months. Survival periods were delineated from the initial admission date extending until either the date of death or the censoring event occurred.

### 2.4 Sample size and missing data

The current model was formulated and reported in accordance with the Transparent Reporting of a multivariate prediction model for Individual Prognosis or Diagnosis (TRIPOD) statement [12]. Because of the retrospective nature of the study, sample size calculation was not conducted. Moreover, there is no universally accepted method for calculating sample size in the development and validation of risk models. Potential candidate variables were selected by clinical expertise and prior published research. Candidate variables selected are all required fields in the data collection system of the CRDC cohort. In cases where an outcome was absent, patient data were omitted from the analysis. As a result, the development cohort had no instances of missing data.

### 2.5 Development of the predictive model

We employed the least absolute shrinkage and selection operator (LASSO) approach to identify the most influential variables from the pool of candidates, aiming to reduce model overfitting [13]. The optimal predictors were identified through cross‐validation. Subsequently, the ultimate model was formulated employing the COX proportional hazard regression approach [14]. Schoenfeld residuals were utilized to test the Cox proportional hazards assumption for each covariate [15] (Figure S1). The cumulative predicted mortality probability for AAV patients at time *t* (months) was computed using the following formula:

(1) $$P_{\mathrm{at}\;\text{time}\;t\;\left(\text{months}\right)}=1-S_{0}{\left(t\right)}^{\exp\;\left(\text{prognostic index}\right)}.$$

In the formula, the prognostic index (PI) was defined as the sum of the products of predictors and their respective coefficients. Specifically, *S*_0_(*t*) = *exp*[−*H*_0_(*t*)] [16].

### 2.6 Model performance and internal validation

To evaluate the predictive performance of this model, Harrell's concordance index (C‐index) [17], the Brier score [18], and calibration curves were employed. We scrutinized the model's performance using the enhanced bootstrap method, recognized as the most effective internal validation procedure across all stages of model development and validation [19]. For this purpose, we generated 200 bootstraps. Furthermore, our objective was to devise user‐friendly measures, accessible to both clinicians and patients, for the stratification of AAV patients into high, medium, or low death risk categories. To achieve this goal, we delineated three risk groups by establishing probability thresholds and anticipated probabilities. This involved implementing stratification cutoff points at the 50th and 75th percentiles of the distribution of probabilities.

### 2.7 Statistical Analysis

Numeric data were represented using the median (interquartile range, IQR), and categorical data were presented as percentages or numerical values. Numeric data were compared using either the independent sample *t* test or the Mann–Whitney *U* test, depending on the distribution. Categorical data, on the other hand, were assessed using the Chi‐square test or Fisher's exact test, as appropriate. The statistical significance was assessed using a two‐sided approach, where *p* values less than 0.05 were considered significant. The analysis was performed using SPSS Statistics for Windows (version 25.0), as well as R software (version 4.3.0).

## 3 Results

**FIGURE 1:**
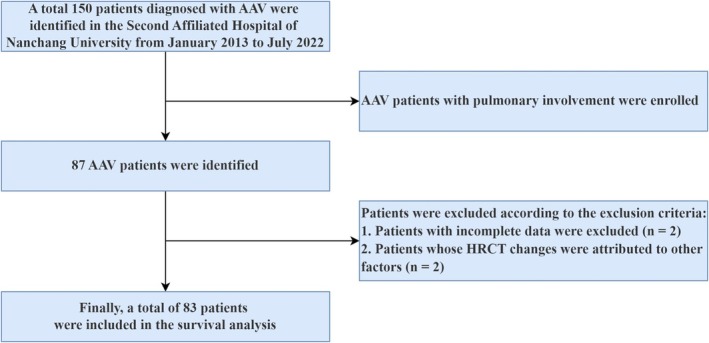
A flowchart illustrating the study design. AAV, antineutrophil cytoplasmic antibody associated vasculitis; HRCT, high‐resolution computed tomography.

Throughout the study duration, a total of 87 patients diagnosed with pulmonary involvement in AAV were included. According to the inclusion and exclusion criteria, two patients with incomplete data were excluded, along with two patients whose HRCT changes were attributed to other factors. Consequently, a total of 83 AAV patients with lung involvement were included in the model, including 15 patients with AH, 38 patients with ILD, 12 patients with PG, and 18 patients with AI (Figure 1). The median follow‐up duration was 7 months (IQR 3–18.5 months). During this period, a total of 12 patients died. The baseline clinical characteristics are presented in Table 1.

**TABLE 1:** Baseline clinical characteristics of pulmonary AAV patients.

Variables	Median (IQR) OR *n* (%)
Male, *n* (%)	44 (53.0)
Age at admission, years	65.00 (50.00, 73.00)
Course, months	32.00 (21.00, 60.00)
Time from onset to diagnosis, months	4.00 (2.00, 13.00)
Smoking history, *n* (%)	27 (32.5)
Hypertension, *n* (%)	20 (24.1)
Diabetes, *n* (%)	12 (14.5)
Coronary heart disease, *n* (%)	4 (4.8)
Tumor, *n* (%)	2 (2.4)
Anemia, *n* (%)	67 (80.7)
Hyperlipidemia, *n* (%)	6 (7.2)
White blood cell (*10^9^/L)	8.72 (6.30, 11.68)
Hemoglobin (g/L)	99.00 (76.00, 119.50)
Platelet counts (*10^9^/L)	261.00 (214.00, 317.50)
Serum albumin (g/L)	32.81 (28.81, 37.61)
Serum creatinine (μmol/L)	85.10 (60.20, 254.56)
Blood urea nitrogen (mmol/L)	7.49 (4.62, 15.56)
eGFR (ml/min)	69.06 (24.77, 105.78)
MPA, *n* (%)	67 (80.7)
GPA, *n* (%)	10 (12.1)
EGPA, *n* (%)	6 (7.2)
MPO‐ANCA(+), PR3‐ANCA(−), *n* (%)	63 (75.9)
MPO‐ANCA(+), PR3‐ANCA(+), *n* (%)	8 (9.6)
MPO‐ANCA(−), PR3‐ANCA(−), *n* (%)	12 (14.5)
ESR (mm/h)	50.00 (20.50, 80.50)
CRP (mg/L)	29.90 (7.84, 84.00)
Immunoglobulin G (g/L)	14.80 (11.40, 17.55)
Immunoglobulin M (g/L)	2.54 (1.80, 3.56)
Immunoglobulin A (g/L)	1.00 (0.67, 1.34)
Complement 3 (g/L)	0.87 (0.75, 0.99)
Complement 4 (g/L)	0.20 (0.17, 0.22)
Alveolar hemorrhage, *n* (%)	15 (18.1)
Interstitial lung disease, *n* (%)	38 (45.8)
Pulmonary granuloma, *n* (%)	12 (14.5)
Airway involvement, *n* (%)	18 (21.6)
General manifestations, *n* (%)	61 (73.5)
Musculoskeletal manifestations, *n* (%)	24 (28.9)
Cutaneous manifestations, *n* (%)	6 (7.2)
Mucocutaneous manifestations, *n* (%)	2 (2.4)
Eye manifestations, *n* (%)	10 (12.0)
Ear nose and throat manifestations, *n* (%)	21 (25.3)
Cardiovascular manifestations, *n* (%)	5 (6.0)
Gastrointestinal manifestations, *n* (%)	0 (0.0)
Renal manifestations, *n* (%)	59 (71.1)
Nervous systemic manifestations, *n* (%)	11 (13.3)
Venous thrombosis, *n* (%)	2 (2.4)
Glucocorticoid pulse therapy, *n* (%)	25 (30.1)
Cytoxan, *n* (%)	58 (69.9)
MMF, *n* (%)	8 (9.6)
Gamma globulin, *n* (%)	5 (6.0)
Plasma exchange, *n* (%)	16 (19.3)
Hemodialysis, *n* (%)	12 (14.5)
Rituximab, *n* (%)	2 (2.4)
FVC%pred	77.90 (55.50, 91.90)
FEV1%pred	78.50 (52.90, 98.15)
FEV1/FVC	87.44 (72.89, 98.03)
MEF75%pred	73.20 (15.80, 114.05)
MEF50%pred	90.00 (30.25, 128.60)
MEF25%pred	62.30 (23.60, 140.30)
MMEF75/25%pred	82.30 (30.60, 129.90)
TLC%pred	72.20 (52.10, 100.20)
DLCO%pred	46.80 (32.20, 69.40)

Abbreviations: %pred, the percentage of the normal predicted value; ANCA, antineutrophil cytoplasmic antibody; BVAS, Birmingham vasculitis activity score; CRP, C‐reactive protein; DLCO, diffusing capacity of the lungs for carbon monoxide; eGFR, estimated glomerular filtration rate; EGPA, eosinophilic granulomatosis with polyangiitis; ESR, erythrocyte sedimentation rate; FEV1, forced expiratory volume in the first second; FVC, Forced vital capacity; GPA, granulomatosis with polyangiitis; IQR, interquartile range; MEF, maximal expiratory flow; MMF, mycophenolate mofetil; MPA, microscopic polyangiitis; MPO, antimyeloperoxidase; PR3, anti proteinase3; TLC, Total lung capacity.

### 3.1 Selection of predictor variables

**FIGURE 2:**
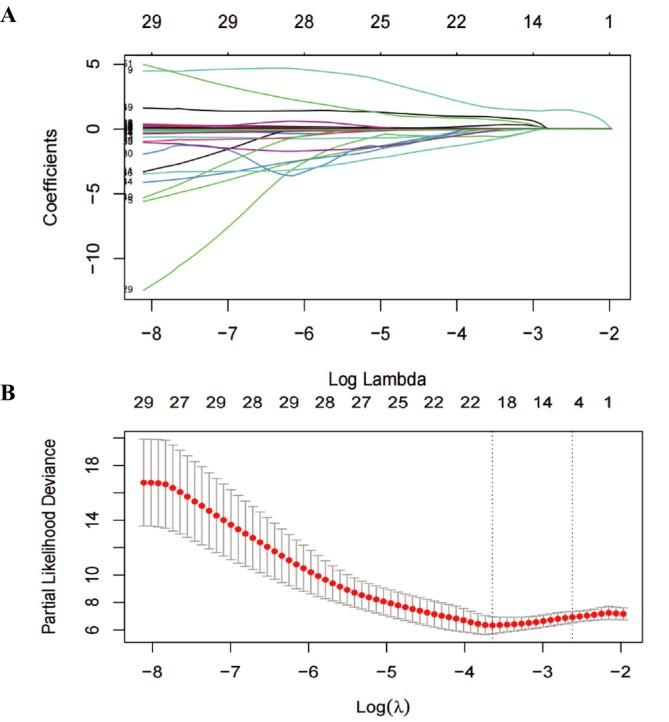
Variable selection was performed using the least absolute shrinkage and selection operator (LASSO) time‐to‐event Cox regression model. (A) Variable selection was conducted utilizing the least absolute shrinkage and selection operator (LASSO). (B) Parameter tuning was accomplished through cross‐validation within the LASSO model.

Five variables demonstrating nonzero coefficients were chosen as predictors for the final model based on the LASSO regression (Figure 2). The final variables were selected according to the results of multivariate COX regression analysis. These predictors consist of age at baseline, history of tumors, baseline hemoglobin level, and the level of the percentage of forced vital capacity to the normal predicted value. The coding and definitions of these four predictors are outlined in Table S1. Additionally, the LASSO coefficients for these predictors in the stacked datasets are provided in Table 2.

**TABLE 2:** The coefficients of the predictors in the final prediction model obtained through LASSO.

Predictors	Coefficient
Age	0.004134029
Tumor	1.482955828
Hb	−0.006353797
FVC%pred	−0.001822463

Abbreviations: FVC%pred, the percentage of forced vital capacity to the normal predicted value at baseline; Hb, hemoglobin.

### 3.2 Model Development

**FIGURE 3:**
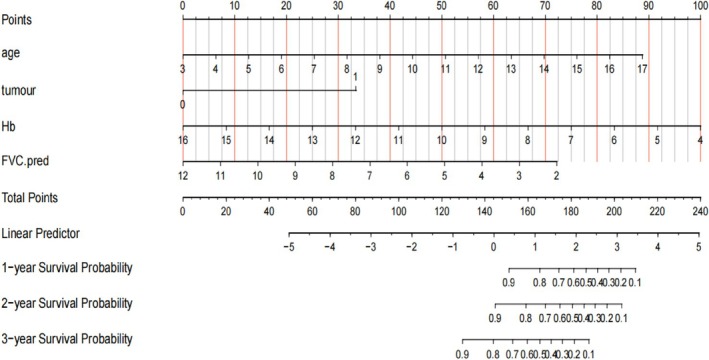
Nomogram for the risk prediction model of AAV with pulmonary involvement.

The entire dataset of follow‐up records, comprising 83 patients with 12 events, was utilized in developing the prediction model. Hazard ratios and corresponding 95% confidence intervals were determined by fitting Cox proportional hazards models (refer to Table 3). The cumulative risk of all‐cause death within a 3‐year period for an individual patient with pulmonary involvement in ANCA‐associated vasculitis (AAV) can be computed using the following formula:

$$P_{\mathrm{at}\;\text{time}\;t\;\left(3\;\text{year}\right)}=1–0.9956803^{\exp\;\left(\mathrm{PI}\right)},$$

where the PI = 0.3335 × Age/5 + 1.7548 × Tumors − 0.4383 × Hb/10–0.3799 × FVC% pred/10. All of the predictors had a positive correlation. A visual representation of this prediction algorithm is depicted as a nomogram in Figure 3.

**TABLE 3:** Prediction model for risk assessment in AAV patients with pulmonary involvement.

Predictor variable	*β* coefficient	HR (95% CI)	*p*
Age	0.3335	1.3958(1.0494–1.8567)	0.02197
Tumor	1.7548	5.7825(1.1458–29.1826)	0.03361
Hb	−0.4383	0.6451(0.4718–0.8821)	0.00604
FVC%pred	−0.3799	0.6839(0.5266–0.8882)	0.00439

Abbreviations: 95% CI, 95% confidence interval; FVC%pred, the percentage of forced vital capacity to the normal predicted value at baseline; Hb, hemoglobin; HR, hazard ratio.

### 3.3 Performance of the Model and Internal Validation

The model's performance in predicting the 1‐, 2‐, and 3‐year death risk for patients with pulmonary involvement in AAV was assessed using 83 patients with 12 events. The prediction model exhibited an apparent C‐index of 0.884 and an integrated Brier score of 0.088, as shown in Table 4. To mitigate overfitting bias, 200 bootstrap samples with replacement were created to calculate the optimism. The corrected C‐index for optimism and integrated Brier score were calculated as 0.850 and 0.085. The C‐index curve varied over time and as did the optimism corrected C‐index over time (Figure S2).

**TABLE 4:** Model performance.

	Current model performance (95%CI)	Average optimism calculated from 200 bootstrap validation	Optimism‐corrected performance
Overall C‐index	0.884(0.811–0.956)	0.034	0.850
Integrated Brier score	0.088	0.003	0.085

Abbreviations: C‐index, concordance index; CI, confidence interval.

**FIGURE 4:**
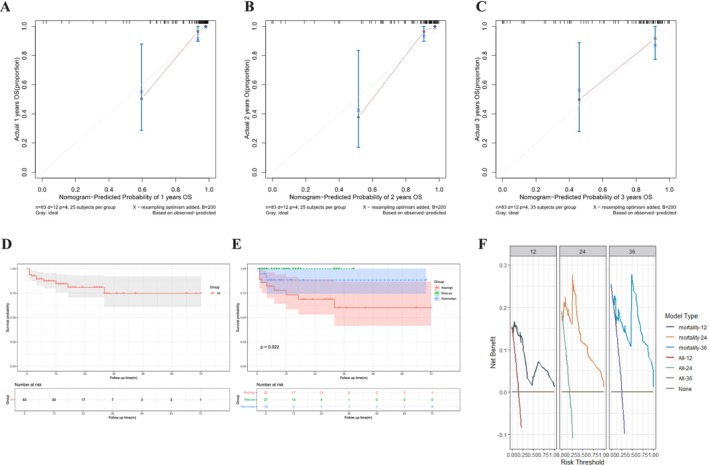
The calibration curve of the AAV with pulmonary involvement, the Kaplan–Meier curves depicting observed survival among AAV patients with pulmonary involvement and the decision curve analysis of the AAV with pulmonary involvement prognostic model. (A) The 1‐year calibration curve of the AAV with pulmonary involvement prognostic model by comparing the observed versus predicted survival. (B) The 2‐year calibration curve of the AAV with pulmonary involvement prognostic model by comparing the observed versus predicted survival. (C) The 3‐year calibration curve of the AAV with pulmonary involvement prognostic model by comparing the observed versus predicted survival. (D) The Kaplan–Meier curves depicting observed survival among all AAV patients with pulmonary involvement to our proposed prognostic model. (E) The Kaplan–Meier curves depicting observed survival among AAV patients with pulmonary involvement in three risk groups according to our proposed prognostic model. (F) Decision curve analysis of the prognostic model for AAV with pulmonary involvement. AAV, antineutrophil cytoplasmic antibody associated vasculitis.

To compare the disparities between actual observed outcomes and predicted risks at 1, 2, and 3 years, we adopted a calibration plot based on 200 bootstrap replications (Figure 4A).

### 3.4 Risk Stratification and Net benefit

Using the tertile ranges of PI in the consolidated dataset, AAV patients were categorized into three risk groups. We categorized these groups as low risk (PI < −3.157), moderate risk (−3.157 ≤ PI < −1.852), and high risk (PI ≥ −1.852). Kaplan–Meier curves were generated by amalgamating outcomes of distinct PI subgroups, and there was a significant difference among the three risk groups (Figure 4B). In the 1‐, 2‐, and 3‐year decision curve analysis (Figure 4C), the prediction model's curve demonstrated a positive net benefit across various probability thresholds.

## 4 Discussion

Patients diagnosed with pulmonary involvement in AAV tend to have a poorer prognosis, risk assessment, and prognosis prediction, which are crucial for them. This study developed and internally validated a prediction model for mortality associated with pulmonary involvement in AAV, utilizing data acquired by the CRDC. Our model incorporates readily obtained clinical risk factors: age at baseline, history of tumors, baseline hemoglobin level, and the level of the percentage of forced vital capacity to the normal predicted value. The established nomogram can predict the 1‐, 2‐, and 3‐year survival probabilities. The internal validation of the model demonstrated its strength and satisfactory performance. According to predicted probability and clinical applicability, patients can be divided into three groups, with specific attention given to the high‐risk group.

The clinical prediction model in the study has several advantages. First, the data were obtained from the CRDC, the largest rheumatology data sharing platform in China, providing high‐quality and reliable data [20]. Second, in contrast to previous studies focusing on renal involvement prognosis in AAV [21, 22], our study emphasized the prognosis of pulmonary involvement in AAV. In addition, our prospective collection of retrospectively analyzed cohort studies can be mined to a greater extent for more possible predictive variables, in contrast to previous case–control or cross‐sectional studies [7]. Third, the model's construction involved the utilization of easily accessible variables combined with a precise formula, facilitating its seamless integration into clinical environments.

In order to ensure the clinical rationality, feasibility, and applicability of the final selection of predictive factors, we sought expert opinions and consulted previous research findings. Internal validation was also conducted to assess their robustness. The final prediction model for death included four clinical variables: age at baseline, history of tumors, baseline hemoglobin level, and the level of the percentage of forced vital capacity to the normal predicted value. In previous studies, multivariate analysis revealed that age over 65 years old at diagnosis and low serum hemoglobin level were identified as independent predictors of mortality [21, 23, 24]. A study specifically comparing factors linked to short‐term prognosis between the elderly and the nonelderly patients with AAV suggests a significant association between age and all‐cause mortality in the elderly cohort [25]. Age is also a component of our final clinical prediction model and is positively correlated with mortality. Therefore, greater attention should be given to elderly patients with AAV lung involvement. Similar to another study on a clinical prediction model for the prognosis of AAV‐related ILD in China, advanced age is often identified as a significant risk factor influencing the prognosis of pulmonary involvement in AAV [26]. However, unlike our findings, the final CASA prediction model in that study also included cardiac involvement, albumin levels, and smoking history as predictive variables. The primary reasons for the differences between our conclusions and the CASA model are as follows. First, the population included in our study differs from that of the CASA model. Our study focused on patients with overall pulmonary involvement in AAV, and the data were derived from China's largest rheumatology data‐sharing platform, the CRDC. Second, China is a geographically vast and demographically diverse country. Regional differences exist between our study and the CASA model study, which may account for the variation in conclusions. These differences provide valuable insights for improving prognostic predictions for AAV patients with pulmonary involvement in both northern and southern regions of China. Due to the prevalence of MPA among AAV patients in our country, often accompanied by renal involvement, anemia is commonly observed in AAV patients and is associated with a higher mortality rate. Low hemoglobin levels serve as an independent risk factor for mortality. AAV patients with lung involvement may also have concurrent renal complications [27]. Consistent with the findings of a study involving 460 patients with AAV and pulmonary involvement, pulmonary manifestations represent a critical factor contributing to mortality in AAV. Furthermore, the presence of concurrent renal involvement, particularly when associated with severe complications such as acute kidney injury, intensive care unit admission, or dialysis dependency, is strongly indicative of a poor prognosis [28]. Furthermore, to a certain extent, anemia is correlated with severe systemic inflammation, further exacerbating the condition of AAV patients with lung involvement and impacting their prognosis [11].

Cancer has been implicated as a possible causal or disease‐triggering factor in AAV. This implies that inflammatory responses triggered by the underlying neoplasm contribute to the pathogenesis of malignancy‐associated vasculitis [29, 30, 31, 32]. Certain instances of vasculitis may manifest in individuals with a history of or simultaneous cancer due to shared risk factors for both conditions [33]. Furthermore, immunosuppressive treatment diminishes the immune system's capacity to identify and eradicate malignant cell clones, and it may possess direct mutagenic properties [34]. Whether it is inflammation triggered by potential tumors or the immunosuppressive treatment that reduces the immune system's capability to recognize and eradicate malignant cell clones, it may potentially impact the prognosis of AAV patients with lung involvement.

The percentage of forced vital capacity to the normal predicted value (FVC%pred), as a key indicator in pulmonary function tests, reflects the vital capacity and lung compliance, making it crucial for assessing lung function and diagnosing pulmonary diseases. FVC% pred is commonly utilized to monitor the condition of patients with AAV‐ILD. Acute exacerbation is one of the causes of death in patients with AAV‐ILD, and FVC% pred is an independent risk factor for acute exacerbation of AAV‐ILD [35]. In our final clinical predictive model, FVC% was included, suggesting its predictive significance in the prognosis of AAV patients with pulmonary involvement, particularly those developing pulmonary fibrosis [36]. Spirometry is a noninvasive, relatively straightforward, and cost‐effective method [37]. FVC measurements offer indications of trends over time in AAV individuals with pulmonary involvement.

Previous studies have suggested that the pulmonary involvement pattern in AAV can influence the prognosis of AAV [38]. Therefore, we categorized patients into four groups based on the radiological pattern of pulmonary involvement in AAV and included it as a filtering variable for further analysis. Indeed, in our earlier study investigation of prognostic factors for pulmonary involvement in AAV, we observed differing outcomes among patients with four distinct patterns of lung involvement. However, the four patterns of lung involvement were not included in the final clinical prediction model. This may be attributed to the relatively small sample size in our study, and the collected data might not have been sufficient to incorporate these factors into the ultimate model. Thus, further exploration through large‐sample, multicenter studies is warranted.

Several limitations exist in our clinical prediction model study. In our study, patients were exclusively sourced from a single center, with a predominant representation of inpatients, thereby potentially introducing patient selection bias. Internal validation of our predictive model was solely conducted through self‐bootstrapping methods. Moving forward, additional research efforts are essential to externally validate our study findings. Therefore, careful consideration must be given to the generalizability of our research results, particularly in relation to other regions and ethnicities. Further investigation is imperative to corroborate the proposed model and measure its performance accurately.

## 5 Conclusions

The proposed risk prediction model in this study provides personalized risk estimates for all‐cause mortality in AAV patients with pulmonary involvement, intended for use by experienced physicians in managing the condition. The long‐term survival prediction model crafted in this study demonstrates strong performance. External validation will be essential to verify the accuracy of this model across various AAV with pulmonary involvement cohorts.

## Author Contributions

All authors participated in the study's conception and design. Qifang Guo drafted the initial manuscript, and all authors provided feedback on previous versions. All authors reviewed and approved the final manuscript.

## Ethics Statement

This study was approved by the Ethics Committee of the Second Affiliated Hospital of Nanchang University (Approval Number 202212).

## Conflicts of Interest

The authors declare no conflicts of interest.

## Supporting information


**Table S1** Definitions of the predictors in the final prediction model.
**Figure S1** Cox proportional hazards assumption for each covariate.
**Figure S2** The c‐index curve over time and the optimism corrected C‐index over time for the risk prediction model of AAV with pulmonary involvement. A, The c‐index curve over time for the risk prediction model of AAV with pulmonary involvement. B, The optimism corrected C‐index over time for the risk prediction model of AAV with pulmonary involvement.

## Data Availability

The datasets used in this study are not publicly accessible because they are also part of an ongoing investigation. However, they can be obtained from the corresponding author upon reasonable request.
