# Supplementary material for: Development of a Nomogram Model to Predict Mortality in ANCA‐Associated Vasculitis Patients With Pulmonary Involvement
Source: Clin Respir J. 2025 Apr 22;19(4):e70067. doi: 10.1111/crj.70067 (PMC12012647; doi:10.1111/crj.70067)

**Supplementary Material**

Analysis of risk factors and development of a nomogram model to predictmortality in ANCA-associated vasculitis patients with pulmonary involvement

Qifang Guo, Yijia Shao, Le Yu, Xiuling Zhang, Jingjing Shang, Xueqin Feng, Rongwei Zhang, Shaoyuan Mao, Haoguang Li, Wei Zhou and Xinwang Duan

**Supplementary Table 1.** Definitions of the predictors in the final prediction model

|  | Definition | Coding |
| --- | --- | --- |
| Age | The age at baseline. It was divided by five in prediction model and in  Nomogram. | Continuous/5 (years) |
| Tumour | AAV associated with tumour based on the international classification of diseases | Binary (no 0, yes 1) |
| Hb | Levels of hemoglobin at baseline. It was divided by ten in prediction model and in Nomogram. | Continuous/10 (g/L) |
| FVC%pred | Levels of the percentage of forced vital capacity to the normal predicted value at baseline. It was divided by ten in prediction model and in Nomogram. | Continuous/10 (%) |

Hb, hemoglobin; FVC%pred, the percentage of forced vital capacity to the normal predicted value at baseline.

**Supplementary Figure 1.** Cox proportional hazards assumption for each covariate


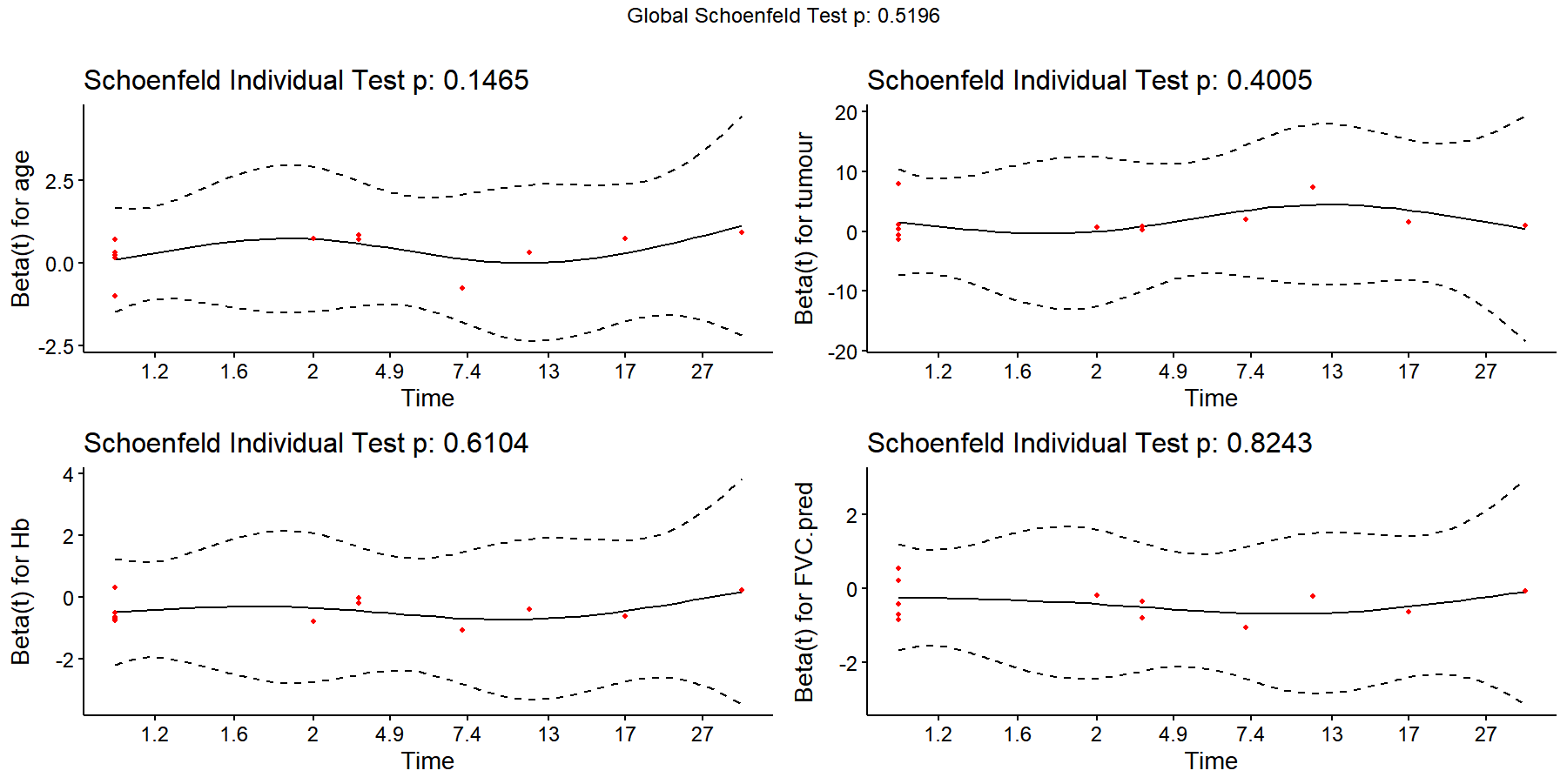


Hb, hemoglobin; FVC%pred, the percentage of forced vital capacity to the normal predicted value at baseline.

**Supplementary Figure 2. The c-index curve over time and the optimism corrected C-index over time for the risk prediction model of AAV with pulmonary involvement. A, The c-index curve over time for the risk prediction model of AAV with pulmonary involvement. B, The optimism corrected C-index over time for the risk prediction model of AAV with pulmonary involvement.**

1.
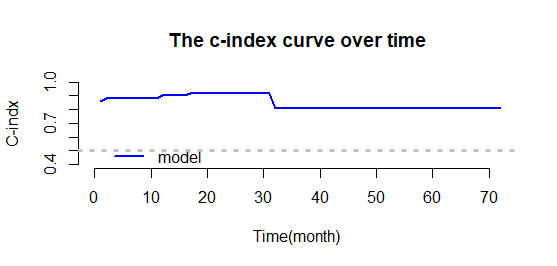

2.
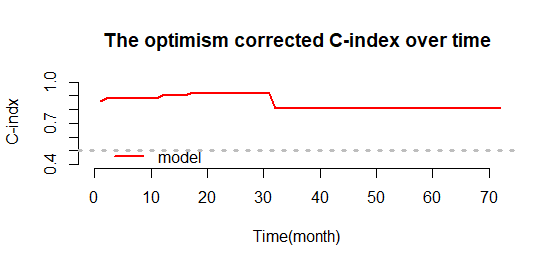

Supplement: Supplementary file 1 — Table S1 Definitions of the predictors in the final prediction model. Figure S1 Cox proportional hazards assumption for each covariate. Figure S2 The c‐index curve over time and the optimism corrected C‐index over time for the risk prediction model of AAV with pulmonary involvement. A, The c‐index curve over time for the risk prediction model of AAV with pulmonary involvement. B, The optimism corrected C‐index over time for the risk prediction model of AAV with pulmonary involvement. [file CRJ-19-e70067-s001.docx]
